# To catch a hijacker: abundance, evolution and genetic diversity of P4-like bacteriophage satellites

**DOI:** 10.1098/rstb.2020.0475

**Published:** 2022-01-17

**Authors:** Jorge A. Moura de Sousa, Eduardo P. C. Rocha

**Affiliations:** Institut Pasteur, Université de Paris, CNRS, UMR3525, Microbial Evolutionary Genomics, Paris 75015, France

**Keywords:** mobile genetic elements, phage satellites, horizontal gene transfer, evolutionary genomics

## Abstract

Bacteriophages (phages) are bacterial parasites that can themselves be parasitized by phage satellites. The molecular mechanisms used by satellites to hijack phages are sometimes understood in great detail, but the origins, abundance, distribution and composition of these elements are poorly known. Here, we show that P4-like elements are present in more than 30% of the genomes of Enterobacterales, and in almost half of those of *Escherichia coli*, sometimes in multiple distinct copies. We identified over 1000 P4-like elements with very conserved genetic organization of the core genome and a few hotspots with highly variable genes. These elements are never found in plasmids and have very little homology to known phages, suggesting an independent evolutionary origin. Instead, they are scattered across chromosomes, possibly because their integrases are often exchanged with other elements. The rooted phylogenies of hijacking functions are correlated and suggest longstanding coevolution. They also reveal broad host ranges in P4-like elements, as almost identical elements can be found in distinct bacterial genera. Our results show that P4-like phage satellites constitute a very distinct, widespread and ancient family of mobile genetic elements. They pave the way for studying the molecular evolution of antagonistic interactions between phages and their satellites.

This article is part of the theme issue ‘The secret lives of microbial mobile genetic elements’.

## 1. Introduction

Bacteriophages (phages) use their bacterial hosts to replicate, which usually ends in bacterial death and the release of virions containing the phage genome. However, phages have their own parasites, satellite mobile elements that cannot produce virions and instead hijack those of functional (so-called helper) phages [1]. The best-studied phage satellites are P4 in *Escherichia coli* [2,3], *Staphylococcus aureus* pathogenicity islands (SAPIs) and phage-inducible chromosomal islands (PICIs) in *Bacillales* and enterobacteria [4], and phage-inducible chromosomal islands-like elements (PLEs) in *Vibrio* spp [5,6]. Their genome sizes vary between 11 kb in P4 and 18 kb in PLE. These values are well below the average genome size of dsDNA temperate phages, which in these clades tend to be larger than 30 kb [7]. Phage satellites integrate the bacterial host genome and may change its phenotype. For example, some SaPIs encode important virulence factors [8], whereas P4 and PLE encode anti-phage defence systems [9,10].

The satellite–helper system that is best understood in molecular terms is the pair P4–P2. P2 is a fully functional enterobacteriophage of the *Myoviridae* family, that can be exploited by the satellite P4. The co-infection of an *E. coli* bacterium with both P2 and P4 provides the context for the parasitism of the latter. P4 was initially regarded as a phage–plasmid, i.e. a phage that can reside in the lysogenic cells as a plasmid [11], and to date there is still no clear evidence on whether P4 is phage, a plasmid or a completely distinct mobile element. However, decades of studies have revealed the key functions of genes encoded by P4 [3,11]. While its *cnr* gene controls the copy number of the plasmid stage of the satellite, P4 also encodes an integrase and is often found integrated in the chromosome. Its transmission through the plasmid life cycle is thought to be infrequent, as only approximately 1% of the P4 infections result in its establishment as a plasmid [12]. Other genes encoded by the satellite are specifically associated with its ability to either replicate or hijack the helper phage. P4 subverts the gene expression of P2 using Ash (also called *ε*), which inactivates the repressor of the helper phage, causing its induction [13]. The P4-encoded *δ* gene is a homologue of P2's *ogr* transcriptional activator and both promote the expression of P2's and P4's late genes [14]. The *α* gene encodes a protein with primase and helicase activities and is essential for DNA replication of P4 [15]. The gene *alpA* is predicted to act as a DNA-binding transcriptional regulator. Finally, the concerted action of the Psu and Sid proteins results in the constriction of P2 capsids and allows P4 to hijack the P2 virion [16,17]. As a result, the modified virions are able to package the DNA from the small genome of P4, but not from the larger genome of P2. Recent structural work has revealed that Sid and Psu are homologues with some sequence similarity and similar folds [18].

Despite the exquisite knowledge obtained in the past decades on the molecular interactions between P4 and P2, we know little about the diversity and distribution of the P4 family of satellites, which also hinders the study of their evolution [19]. P4-like elements may be difficult to identify using prophage identification tools because they lack typical distinguishing phage components, like packaging or virion proteins, and the diversity of their gene repertoires is unknown. Nevertheless, a few studies have suggested the existence of other P4-like elements. For instance, a cryptic P4-like prophage was found to excise and express an AlpA homologue [20], and another one, encoding a retron, was shown to require P2 for transfer [21]. Additionally, a study of prophage domestication identified 28 elements with a homologue of *sid* [22], and a recent study identified more than 5000 homologues of *psu* in *ca* 20 000 *E. coli* genomes [9].

These studies raised key questions. Is P4 part of a family of mobile genetic elements with conserved gene repertoires and genetic organizations? What is the core genome and the genetic organization of such a family? Are members of the family abundant in bacterial genomes? Have P4-like elements recently derived from other mobile genetic elements, e.g. phages, or are they ancient? To provide answers to these questions, we searched bacterial genomes for regions with clusters of homologues to the abovementioned key components of P4. Given the lack of available methods to detect P4-like satellites, we studied the composition of these clusters to uncover and characterize a putative family of P4-like satellites. We quantified their abundance, which resulted in the identification of *ca* 1000 novel P4-like mobile elements. This allowed us to study their prevalence and association with bacterial hosts. We also characterized the composition and organization of the gene repertoires of P4-like elements, as well as the phylogenetic history of key genes. Our results uncover the hidden diversity of P4-like satellites and highlight their role as a distinct mobile element in the microbial world.

## 2. Results

### (a) P4-like satellites are a large and well-defined family of mobile elements

The genetic diversity of P4-like satellites is currently unknown, which complicates their identification. We started our study by searching for genomes related to P4 among the 2487 sequenced phages from the NCBI RefSeq database, using the weighted Gene Repertoire Relatedness index (wGRR, see Methods), a measure of the proteome similarity between pairs of genomes. This failed to reveal P4-like elements because 93% of the phages are completely unrelated to P4 (wGRR = 0, electronic supplementary material, figure S1A), while the remaining 7% show very low wGRR (less than 0.06). The analysis of sequence similarity between P4 proteins and all the proteins in the phage database revealed hits with an *e*-value higher than 10^−5^ for the integrase, the replicase and the transcriptional regulators. Delta proteins showed low homology (all hits with less than 40% identity) with a few P2-like genomes. Moreover, among the 26 984 prophages detected in 13 513 bacterial genomes, less than 0.1% have a wGRR with P4 above 0.1 (electronic supplementary material, figure S1B). The few prophages with high wGRR consisted of a P4-like element close to a large prophage. We conclude that there are no phages and very few prophages with extended homology to P4. Hence, either P4-like elements are extremely rare or they are missed by current computational approaches for phage identification.

**Figure 1.**
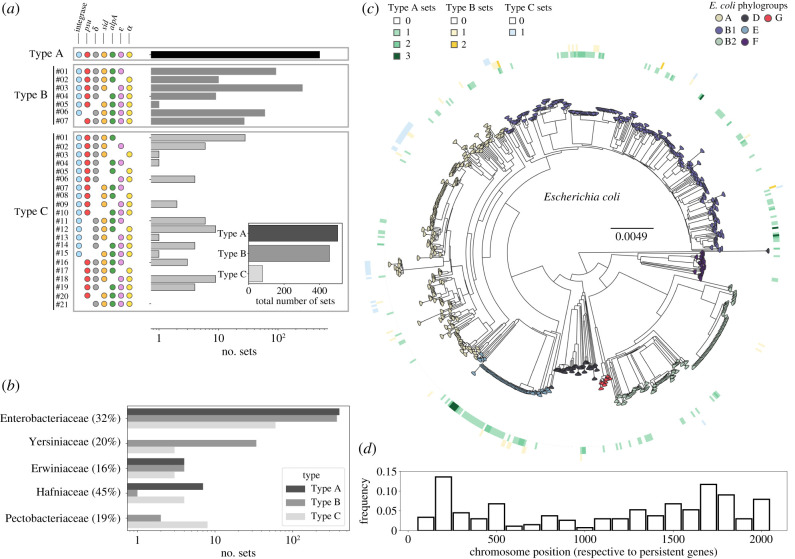
The abundance, genetic composition and bacterial hosts of P4-like elements. (*a*) Log-scale abundance of the variants, defined by the combinations of core components (circles to the left of the distributions). The inset shows the total abundance per type, considering all variants. (*b*) Distribution of the different sets in bacterial families. The percentages following the families indicate the fraction of bacterial genomes of that family that encode P4-like satellites. (*c*) Distribution of P4-like sets in the *E. coli* species tree. The tree was built using maximum likelihood, using the alignment of the core genome (2107 genes) from 657 *E. coli* genomes obtained using PanACoTA [23] and FastTree with default parameters [24]. The presence and abundance of sets of Type A (green), B (yellow) or C (blue) are shown in the inner, middle and outer circle, respectively, with genomes that encode for more than one set of either type shown as a stronger shade of their respective colour. (*d*) Position of the P4-like element along the chromosome of *E. coli.* The core genes of the species were ordered and numbered in function of the position of the gene in the chromosome of strain MG1655 from 1 to 2107. Positions between core genes define genome intervals. The position indicates the interval where the P4-like element was identified. Hence, an element integrating the same region across genomes would have the exact same position.

We used publicly available and custom-built hidden Markov models (HMM) profiles to search for homologues of P4 key components—integrase, *psu, δ*, *sid*, *alpA*, *ε* and *α*—in bacterial genomes, including 11 806 plasmids (see Methods). We ordered these homologues by their genomic location and clustered them in *sets*: groups of co-localized homologues to these components (less than 10 genes apart; electronic supplementary material, figure S2). Since we were unaware if these components would be present in all elements, we allowed sets to miss one or two components. We found 1037 sets (electronic supplementary material, file S1), among which 502 contain all the seven components of P4 (hereafter referred to as Type A, figure 1*a*), 456 lack one component (Type B) and only 79 lack two (Type C). Each possible combination of missing components was deemed as a variant of each type (figure 1*a*). Among the Type B sets, most (56%) lack an homologue to *alpA* (TypeB#03). Variants lacking *α* (TypeB#02) or *psu* are also frequent (TypeB#06). This suggests that these components could be either facultative (i.e. missing from the elements, or present as pseudogenes that do not produce viable proteins), or that they could be replaced by functional homologues in some P4-like elements. On the other hand, only one variant missing *δ* (TypeB#05) was found, suggesting that this is a conserved and essential component of functional P4-like satellites. The most abundant Type C variant lacks both *ε* and *α* (TypeC#01). Notably, seven of the 21 possible variants of Type C were never found. The abrupt decline in the abundance of Type C elements, relative to Types A and B, suggests that there is a clear distinction between elements with a common set of key components homologous to P4 and the other mobile genetic elements.

There has been a longstanding uncertainty concerning the relevance of the plasmid stage in P4 [11,25]. This prompted us to analyse whether the sets we identified—Types A, B or C—are located in bacterial chromosomes or in plasmids. All sets were found in bacterial chromosomes. Although we found some isolated components or rare sets of two components (one being often an integrase) in plasmids, we could not find one of these elements with more than three P4-like components. This suggests that P4-like satellites are not plasmids under normal physiological conditions.

We then analysed the taxonomic distribution of the bacteria with P4-like elements (Types A, B and C). The genomes of Enterobacteriaceae include 92% of the elements, and these are found in 32% of their genomes (figure 1*b*; electronic supplementary material, figure S3). Most genomes have one element, but some have up to three (figure 1*c*; electronic supplementary material, figure S4). The most represented species in our genome dataset is *E. coli*. In this species, 44% of the genomes (out of 657) encode at least one P4-like element. These elements are dispersed and prevalent across the species tree (figure 1*c*). To confirm that their prevalence is not the result of a single ancestral infection, we analysed the positions of P4-like elements in the chromosomes of the species (see Methods). We located the elements in relation to the positions of the neighbouring core genes and found that P4-like elements are scattered across the *E. coli* chromosome (figure 1*d*). The dispersion of the elements across the phylogenetic tree and across the chromosome shows that these elements have proliferated by horizontal transfer across the species.

We also found P4-like elements in genomes of bacterial families that are poorly represented in the genome database: Yersiniaceae (4% of the elements), Hafniaceae (1.3%), Erwiniaceae (1.2%) and Pectobacteriaceae (1%). Interestingly, the P4-like elements in the Yersiniacea family are exclusively comprised Type B that lack a *psu* homologue (TypeB#06). Even though estimates of the prevalence of P4-like elements in these poorly sampled bacterial families are less reliable, given the few genomes available for some families, our analysis reveals that between 16% and 45% of the genomes of each family encode at least one element (figure 1*b*). In summary, P4-like satellites are a very abundant and characteristic mobile element, widespread in the genomes of Enterobacterales.

### (b) Genomic characterization of the P4-like satellite family

**Figure 2.**
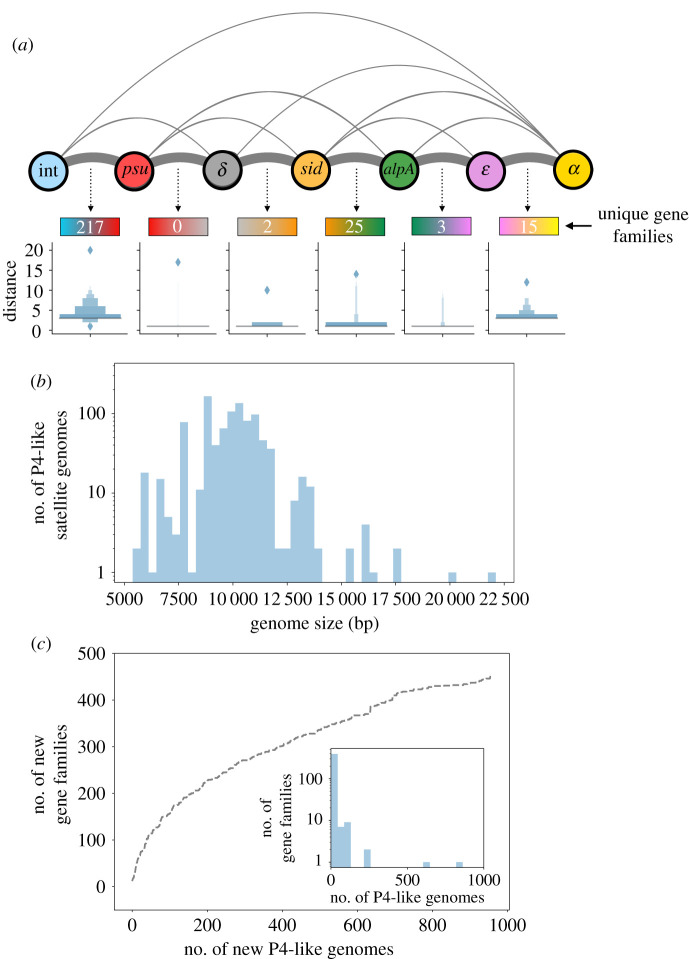
The genomes and pangenome of P4-like satellites. (*a*) Genetic organization of the P4-like elements. Nodes indicate core genes and edges link the nodes when the two core genes are consecutive in the genome organization of core genes. The thickness of the edges between nodes is proportional to the frequency of adjacency of pairs of the corresponding components. The number of unique gene families detected between each two genes are indicated below the space between each respective circle. These families were calculated considering only Type A elements that have the most common organizational variants. The box plots show the distribution of the number of open reading frames between consecutive components. The outliers of these distributions represent the rare organizational variants where the most frequent pairs of components are further than 10 genes apart. (*b*) Histogram of the genome size of P4-like elements. (*c*) Rarefaction curve of the gene families of P4-like genomes, excluding those of core components. New gene families were sequentially added from randomly ordered P4-like genomes. The inset shows the frequency of the gene families.

To study the genetic organization of P4-like elements, we analysed the order of the components using the integrase as a starting position. These analyses were restricted to Types A and B, which comprise approximately 92% of the P4-like satellites and are more likely to include functional elements. The order of the components is very similar for Types A and B, and 77% of the former have the organization of P4 (figure 2*a*; electronic supplementary material, figures S5 and S6). The most frequent exception concerns a swap in the order between *psu* and *δ* (20% of the total), while in a few others the homologues of *alpA* follow *α*'s homologues (1.2%). In very rare cases (less than 1%), the integrase appears next to *α*, and the order of the rest of the components is reversed relative to P4. Such cases seem to reflect the presence of other mobile genetic elements encoding their own integrase and neighbouring P4-like elements whose integrase is either missing or more than 10 genes apart from *psu*. The distance between consecutive components is diverse (figure 2*a*, boxen plots). Some of the core components, like the operon formed by *psu*, *δ* and *sid*, tend to be adjacent, while other consecutive components are several genes apart. For instance, the number of genes between the integrase and *psu* ranges from one to nine, if we consider only the variants where the integrase precedes *psu*. Overall, these results suggest that the organization of the P4-like genome is very conserved.

Because the core components are nearly ubiquitous and organized similarly, we can use the integrase and *α* homologues to tentatively delimit P4-like elements. In the circularized plasmid representation of P4, these two genes are separated only by the *att* site, with no other genes between them. We could not find prevalent gene families (i.e. present in more than 25% of the elements) in the five genes before the integrase or the five genes after *α*, across all sets, showing that if there are satellite genes in these regions, then they are not conserved. For the few Type B sets lacking *α*, and for the rare organizational variants where *α* is not the last component in the set, we used the last component to delimit the satellite genome. While we cannot exclude that some elements may be larger, our results suggest the two components are suitable to delimit the P4-like satellites.

The genomes of P4-like satellites have a median of 10 kb (figure 2*b*), close to the 11 kb of P4, encoding *ca* 11 proteins (electronic supplementary material, figure S7). However, there is considerable variation in their sizes. Some elements have close to 5 kb, whereas a very small number (11 genomes) are more than 15 kb long. The smaller genomes mostly correspond to Type B variants lacking either the integrase or *α*, and were delimited by *psu* and *ε*, which may justify their small size. The removal of these cases raises the minimal genome to approximately 7 kb (electronic supplementary material, figure S8), but still leaves considerable variation in size. The largest elements are often tandem elements or groups of genes encoding homologues of P4 components adjacent to complete P4-like elements. They may reflect the pseudogenization of tandem elements.

The conservation of the repertoires of core components and the large variation in size of the elements suggests high diversity of non-core components. We computed the pangenome of the P4-like family to assess this point more precisely. After removing the core components mentioned above, the gene family frequency spectrum is L-shaped with most gene families (381, 85%) present in fewer than 10 genomes. This abundance of low-frequency gene families results in an open pangenome (figure 2*c*), suggesting that P4-like elements have a large gene repertoire. The estimation of its full size and diversity will require sequencing further genomes in a more varied range of species: 84% of the gene families from P4-like elements do not match phage-like gene profiles with known functions, and 91% of these same gene families also do not match any profile with known bacterial-like functions. Hence, we cannot use sequence similarity to assign functions to the majority of non-core genes of P4-like satellites. However, a few gene families are associated with functions that are relevant to bacterial lifestyle, from defence mechanisms to cell motility (electronic supplementary material, file S2). We then computed the gene families specific to the locations between each pair of core components. We restricted this analysis to genomes of Type A with the most conserved genetic organization to characterize the local pangenome using only the common pairings of adjacent components. Most gene families observed between consecutive core components are located between the integrase and *psu* or between *sid* and *alpA* (figure 2*a*, numbers below organization diagram). The first region corresponds to the previously described hotspot of defence systems [9] and includes the *cos* site in P4. The region between *ε* and *α* has two frequent gene families. One is present in 90% of the elements and includes the *cnr* gene of P4. The other is present in 61% of the elements and encodes a small protein (75 aa) of unknown function that is absent from P4. This suggests that P4 lacks a protein that is prevalent in its family of satellites.

The conserved genetic organization of P4-like elements facilitates the search for pseudogenes or the missing components in some Type B variants. When genes were missing, we analysed the genomic positions where they were expected. We found that most (98%) *alpA*-less variants (TypeB#03) have an *alpA* pseudogene. Their analysis revealed the presence of frameshifts resulting in early stop codons. These events seem to have occurred more than once in different members of the family (and in different bacterial genera) because we find *alpA* pseudogenes in distinct parts of the evolutionary tree of the gene (electronic supplementary material, figure S9). This suggests that *alpA* inactivation is frequent. On the other hand, *psu*-less variants (TypeB#06) reveal at the expected location very diverse genes, and only rarely (8%) a *psu* pseudogene. We were not able to assess the other Type B variants, either owing to their small number of P4-like elements or the uncertainty regarding the expected position of the missing components. Nevertheless, an analysis on the association of accessory genes (located anywhere within the satellites' genomes) with specific missing components suggests that some type B variants are positively correlated with the presence of certain genes (electronic supplementary material, figure S10 and table S1). Namely, variants lacking *psu* (TypeB#06), *δ* (TypeB#05), *ε* (TypeB#02) or *α* (TypeB#01) are significantly enriched for one, and up to four, accessory genes. On the other hand, variants lacking *alpA* are not exclusively associated with specific accessory genes, which suggests that they do not possess functional replacements for this core function.

**Figure 3.**
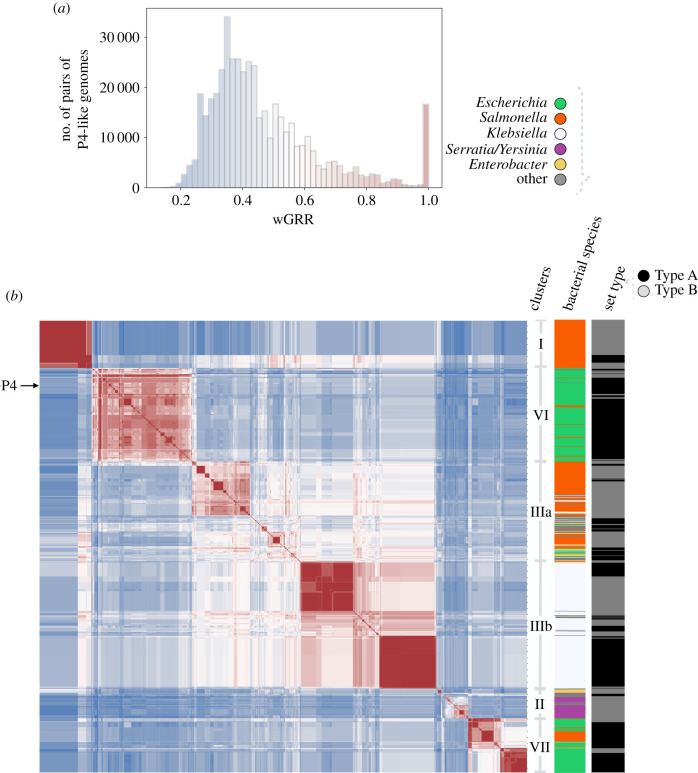
Comparison of gene repertoires of P4-like satellites. (*a*) Histogram of wGRR for all pairwise comparisons between the 958 P4-like genomes (of Types A and B). (*b*) Heatmap of the matrix of the wGRR values ordered using hierarchical clustering. The colours follow the same code as in (*a*). The columns to the right of the heatmap indicate the bacterial species where the P4-like genome was detected and the Type (A or B) of the P4-like genome. The position of the P4 genome in the matrix is indicated on the left side. The clusters were determined by using ‘maxclust’ and a threshold of seven flat clusters as parameters. They are shown as the most common cluster representatives in each main region of the heatmap (the complete detailed list of cluster assignments is given as electronic supplementary material, file S4).

To compare the gene repertoires of P4-like elements, we computed the wGRR between them. Most comparisons (81%) revealed wGRR values between 0.6 and 0.2, suggesting that most P4-like genomes have considerable genetic differences in spite of the core genes present in most elements (figure 3*a*). The peak at wGRR close to one represents pairs of elements that are practically identical and may result from vertical descent or recent transfer across bacteria. We used hierarchical clustering to identify groups of similar satellites in the wGRR pairwise comparisons matrix. The large groups contain both Type A and B elements, but within these main clusters, the different types tend to be grouped together, suggesting the existence of relatively recent lineages with different repertoires of core components. The largest clusters are those of satellites detected in *Escherichia* (clusters VI and VII), *Klebsiella* (cluster IIIb) and *Salmonella* (clusters I and IIIa). P4-like satellites detected in the *Yersiniaceae* family (all from Type B) also form the largest part of a different cluster (cluster II). This suggests that elements tend to be more similar as a function of their bacterial host species or genus. Nevertheless, 4% of the pairwise comparisons with high wGRR (greater than or equal to 0.9, *n* = 19 961) concern elements present in different bacterial species, often in different genus. Furthermore, 14% of the 5731 pairs of elements with average nucleotide identity higher than 95% (the threshold typically used to separate bacterial species [26]) are found in different bacterial species. This suggests that P4-like satellites can disseminate across distantly related bacteria.

### (c) Evolution of P4-like satellites

**Figure 4.**
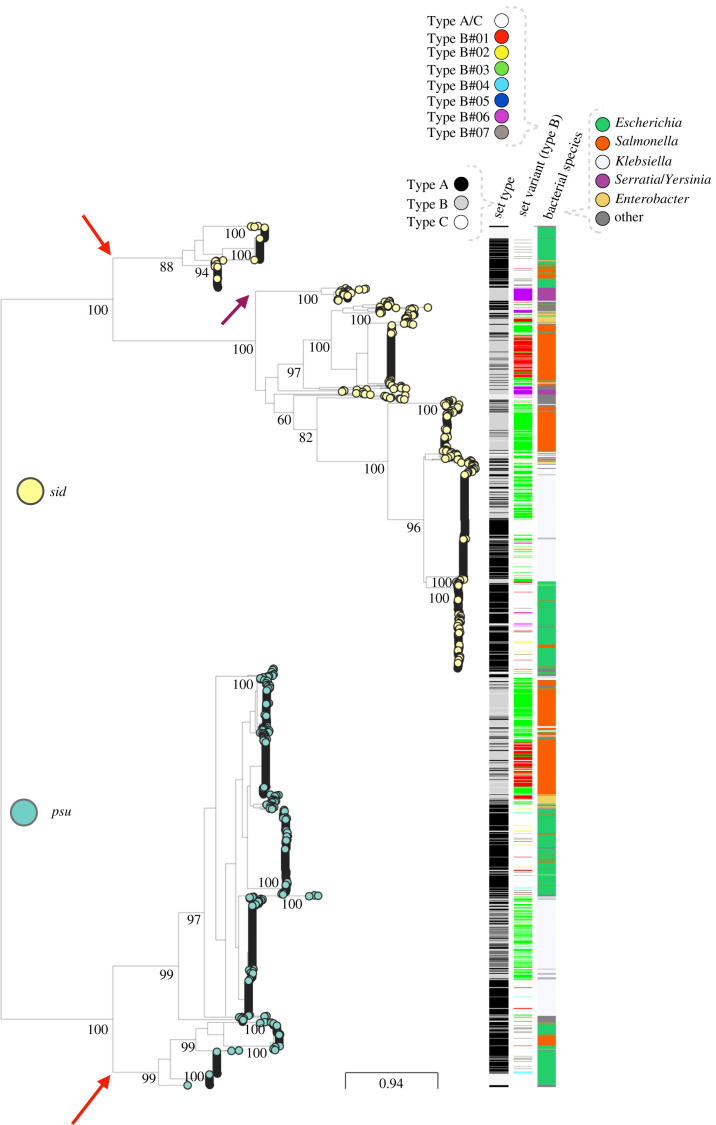
Joint phylogenetic tree of Psu and Sid proteins. The values shown on the branches are the result of ultrafast bootstraps and show a good support for the most important nodes. The columns on the right indicate, from left to right, the Type of set (A, B or C), the variant (only shown for sets of Type B, with Type A and all Type C variants shown as white spaces) and the bacterial species. Red arrows indicate the early branching clades that correspond to cluster VII in figure 3*b*. Purple arrow indicates the clade corresponding to P4-like elements detected in *Serratia* and *Yersinia* bacterial hosts, that are absent in the Psu sub-tree. The Newick tree file is included as electronic supplementary material, file S5.

The genes *psu* and *sid* encode key functions for the hijacking of P2 virions by P4. These genes are structural homologues and share some sequence similarity [18], which suggests they are derived from the same ancestral protein. In such cases, the joint phylogeny of two proteins allows rooting of the sub-tree of each of them [27]. To investigate the evolutionary history of *psu* and *sid*, we aligned their proteins from sets of Type A, B and C and used it to infer the phylogeny. The resulting tree (electronic supplementary material, file S5) is well supported at most key nodes and shows two distinct monophyletic clades that correspond to either Psu or Sid (figure 4). Hence, it can be used to understand the evolution of the two proteins and to distinguish ancestral from derived states. We have also built phylogenetic trees for *δ* and the integrase and computed the patristic distances (i.e. the sum of the lengths of branches that link two nodes in a tree) between the proteins of each family. The correlation between patristic distances of the four protein families shows diverse patterns (electronic supplementary material figure S11, files S6–S9). Notably, the Spearman correlations between the patristic distances of the integrase and those of the other components are very low (between 0.01 and 0.16), showing that this protein has a very different evolutionary history among P4-like elements. The correlations between *δ*, Psu and Sid are much higher, especially those between the two latter proteins (*ρ* = 0.76), revealing joint evolution within the elements.

The sub-trees of Psu and Sid in the large joint phylogeny show a small early branching clade (annotated with red arrows) from elements present in *E. coli* and *S. enterica* that are those from cluster VII in the wGRR matrix (figure 3). The Sid sub-tree then reveals a subsequent clade present in *Serratia*/*Yersinia* (annotated with a purple arrow) that is absent from the Psu tree. Hence, the ancestor of all P4-like satellites already contained the two key genes involved in the parasitism and elements of this clade lost *psu*.

The P4-like genomes of Types A or B are interspersed in the tree, often forming small separate clusters. This is consistent with the observations in the wGRR matrix and suggests that loss of one gene, typically *alpA*, still results in functional elements. The *psu* and *sid* homologues from Type C sets are either dispersed in the tree or clustered at the earlier branching clades of the sub-trees. The former may be genetically decaying P4-like satellites, but the latter might be part of different elements that have long diverged from P4 and lost *ε* and *α*. The phylogenetic tree is also consistent with the analysis of the wGRR matrix in that large clades of closely related proteins include a majority of elements from one bacterial genus and a few elements from other genera. This reinforces the conclusion that these elements can transfer across large taxonomic groups of bacteria.

## 3. Discussion

Recent experimental work has revealed the existence of phage satellites in Proteobacteria and Firmicutes [1]. Satellites of eukaryotic viruses are also known [28]. Such elements are thus likely to exist in other bacterial clades. There was some previous evidence of genetic diversity in PICI, for which more than 20 elements were described across diverse bacterial taxa [4]. Similarly, five different PLE were identified in *Vibrio cholerae* [10]. Yet, there was very little quantitative information on the frequency, gene repertoire diversity and organization and on the taxonomic distribution of phage satellites in Bacteria. Here, we identified more than one thousand P4-like satellites across several genera of Enterobacterales. They constitute a large family of characteristic mobile elements, with distinctive gene repertoires and a highly conserved genetic organization. The most sequenced bacterial species contain numerous such elements, e.g. they are found in almost half of the *E. coli* genomes, sometimes in multiple separate occurrences. These elements show very little homology with phages and are never found as plasmids. Hence, P4-like satellites are neither phages nor plasmids, but instead a distinct and widespread lineage of integrative mobile genetic elements.

Some P4-like genomes miss one or, in rarer cases, two core genes raising the possibility that they are defective. Many prophages are non-functional in bacteria [29,30] and it is possible that some of the P4-like satellites are also non-functional. Yet, three pieces of evidence suggest that most Type B and C sets are functional variants of P4. First, some genes are almost always present whereas one, *alpA*, is more often absent. This gene might be a gene expression regulator [4], which is often replaced in mobile elements. If Type B and C elements were non-functional, one would expect a more random distribution of absent genes. We found that one gene present in most P4-like elements is lacking in P4, in agreement with the idea that highly frequent genes can be missing in functional elements. Second, for at least one Type B variant (those lacking *psu*), the position of the missing component rarely contained a pseudogene of the missing gene (8% of *psu*-less variants). This suggests that at least some of these elements are not recent loss-of-function variants. The case of *alpA* is more complex because its pseudogene is frequent and conserved across some clades. This suggests that either the pseudogenized form provides some type of advantage, e.g. a partial function, or at least that this gene is not essential. This is also supported by the lack of other accessory genes specifically associated with these variants. Third, many of the Type B and C elements form small conserved clades within the Psu + Sid phylogenetic tree, suggesting that they are functional. One particularly intriguing case concerns the elements that have lost *psu*, including those among *Yersineacea*. It is possible that *psu* is not strictly necessary in some clades. Yet, we could not identify specific features to those elements, or their Sid proteins, that could explain the absence of *psu* Overall, while we cannot ascertain the function of all elements that we have identified (or assess the functional similarity of the proteins found in the locations of the missing components or elsewhere in the satellites’ genomes), many seem to contain a full or nearly full repertoire of the key components of the family and may, therefore, be functional.

The origins of P4-like elements are unknown. We observed very little sequence similarity between phages and P4-like elements, suggesting they had distinct evolutionary origins [19]. Most of these homologues concern genes that are frequently transferred across mobile genetic elements like replicases, integrases and transcriptional regulators. Accordingly, the integrase phylogenetic tree is very different from the others. This suggests frequent gene exchanges with other mobile genetic elements and is consistent with the observation above that the elements tend to be scattered across the genome. This is also consistent with the proposal that the *cos* site, between the integrase and *psu*, is associated with rapid diversification of the defence systems carried by P4-like elements [9]. *δ* is the only specific P4 gene that has clear homologues in the *ogr* genes of P2-like phages, but sequence similarity is very low [3]. P2-like phages are probably also very ancient [31] and may have co-evolved with P4-like elements for a long time. The evolutionary history of the two most distinctive P4 genes—*psu* and *sid*—should be key to trace the origins of P4. However, these genes do not show significant similarity with phage genes and while Psu and Sid have similar protein structures, they are very dissimilar from all other known protein structures [18,32]. It is tempting to speculate that *psu* and *sid* originated by gene duplication [18] (or gene transfer leading to an equivalent outcome) followed by specialization into a protein that forms a transient external scaffolding cage around the P4 procapsids (Sid) and another acting as a stabilizing protein at the outside of P4 capsids (Psu). Given the genetic diversity of the components of P4-like elements, their variation in gene repertoires, their broad distribution across Enterobacterales and the differences between Psu and Sid, P4-like elements are probably a very ancient family of phage satellites. Moreover, since *psu* and *sid* are structural homologues, *psu*-less elements could rely on *sid* variants that more closely resemble the putative original, unduplicated gene and could thus be able to single-handedly sequester the capsid of helper phages.

The impact of P4-like satellites depends on their ability to disseminate, the traits they carry and how they affect the helper phages. The high incidence of very similar P4-like satellites in different bacterial genera suggests they have a broad host range. If the host range of the satellite depends on the ability of the virion to attach and inject DNA in a recipient cell, then it should be limited by the host range of its helper phage. However, satellites can increase their host range if they can exploit multiple helper phages. This may be facilitated by the acquisition of genes at the elements' hotspots, which have very diverse gene repertoires contributing to the open pangenome of P4-like elements. These regions also encode traits that affect bacterial fitness, such as the anti-phage defence systems identified in a P4 hotspot [9]. In order to better understand the basis for the variation and distribution of P4-like satellites in bacterial species, we will now analyse their co-incidence with P2-like prophages. This will pave the way to study the population and coevolutionary dynamics of satellites and their helper phages. Our novel collection of P4-like elements will thus facilitate the study of the impact of phage satellites in the diversification of bacterial genomes and in phage–bacteria dynamics.

## 4. Material and methods

### (a) Data

We retrieved the complete genomes of 13 513 bacteria, 11 806 plasmids and 2502 phages from NCBI non-redundant RefSeq database (ftp://ftp.ncbi.nlm.nih.gov/genomes/refseq/, last accessed in May 2019, electronic supplementary material, file S10). Five phage genomes were excluded from the analysis because of lack of gene annotation, resulting in a dataset with 2487 phage genomes. Prophages (integrated temperate phages) were predicted in bacterial genomes using VirSorter v. 1.0.3 with the RefSeqABVir database [33]. The least confident predictions, i.e. categories 3 and 6, which may be prophage remnants or erroneous assignments, were excluded from the analyses, resulting in a total of 26 984 prophages.

### (b) Detection of P4-like elements using reference components

We used HMMER (v. 3.1b2 [34]) to search for homologues of each component (PFAM or custom profiles in parenthesis): Psu (PF07455.12), *δ* (PF04606.13), Sid (custom profile, in the electronic supplementary material, file S11), AlpA (PF05930.10), *ε* (PF10554.10), *α* (PF03288.17) and integrases (PF00589.20, for tyrosine recombinases, and PF00239.19 and PF07508.11 together for serine recombinases) in bacterial and plasmid sequences. We retained the hits with an *e*-value of at most 10^−5^ and a profile coverage of at least 40%. We then ordered the genes by their position and searched for consecutive pairs of components less than 10 open reading frames (ORFs) apart (see the electronic supplementary material, figure S2). Additional components were added by transitivity when they were within 10 ORFs of the set. This was repeated until either the following component was at a distance larger than 10 ORFs (which would start a new process of constructing a different set, closing the current one), or when there were no more components. The sets sometimes contain duplicates of a given component, particularly integrases which tend to aggregate in bacterial genomes at chromosome hotspots [35]. When more than one integrase was part of the set, we kept the integrase closest to *psu*, as expected from the linearization of the original P4 genome, and our analyses confirm the conservation of this location for the integrase. When components were in multiple copies, which was very rare, we kept the first occurrence.

### (c) Pangenomes of the P4-like satellite family

P4-like satellites of Types A and B were delimited by the integrase and the furthest component of the set (in the case of the variant TypeB#07, which lacks the integrase, we used *psu* because it is the component most frequently found after the integrase). We computed the pangenome of the P4-like elements by clustering all their proteins at a minimum of 40% identity, using mmseqs2 [36] (Nature Biotechnology release, August 2017), with parameters –cluster_mode 1 and –min_seq_id 0.4 (all other parameters were left as default). The resulting gene families were functionally annotated using PFAM (release 33.1), bactNOG [37] or pVOG [38]. For the latter, we used a functional categorization of profiles as described previously [39].

### (d) Weighted gene repertoire relatedness between P4-like genomes

We searched for sequence similarity between all proteins of all phages using mmseqs2 (Nature Biotechnology release, August 2017 [36]) with the sensitivity parameter set at 7.5. The results were converted to the blast format and we kept for analysis the hits respecting the following thresholds: *e*-value lower than 0.0001, at least 35% identity and coverage of at least 50% of the proteins. The hits were used to retrieve the bi-directional best hits between pair of phages, which were used to compute a score of gene repertoire relatedness weighted by sequence identity:

$$\mathrm{wGRR}=\frac{\sum_{i}^{p}\mathrm{id}(A_{i},B_{i})}{\min(A,B)},$$

where *A_i_* and *B_i_* is the pair *i* of homologous proteins present in *A* and *B*, id(*A_i_*,*B_i_*) is the sequence identity of their alignment, and min(*A*,*B*) is the number of proteins of the genome encoding the fewest proteins (*A* or *B*). wGRR is the fraction of bi-directional best hits between two genomes weighted by the sequence identity of the homologues. It varies between zero (no bi-directional best hits) and one (all genes of the smallest genome have an identical homologue in the largest genome). wGRR integrates information on the frequency of homologues and sequence identity. For example, when the smallest genome has 10 proteins, a wGRR of 0.2 can result from two homologues that are strictly identical or five that have 40% identity. The hierarchical clustering of the wGRR matrix, and the corresponding heatmap, were computed with the *clustermap* function from the *seaborn* package (v. 0.9, developed for Python 2), using the *average* (UPGMA) clustering algorithm.

### (e) Joint phylogenetic tree of *psu* and *sid* components

We aggregated in a single fasta all protein sequences of *psu* and *sid* from all sets of Types A, B and C, including the respective components from the original P4 genome (NC_001609). The sequences were aligned using mafft-linsi [40] (v. 7.222, default parameters) and the resulting alignment trimmed with noisy [41] (v. 1.5.12, default parameters). We used IQ-Tree [42] (v. 1.6.5) to build the phylogenetic trees, with the options –wbtl (to conserve all optimal trees and their branch lengths), –bb 1000 to run the ultrafast bootstrap option with 1000 replicates, -m MFP for automatic model selection and –nt AUTO. The resulting tree files were converted to Newick format using the Phylogeny.fr [43] webserver (http://phylogeny.lirmm.fr/phylo_cgi/index.cgi, last accessed March 2021). The visualization of the tree was performed using the Microreact [44] webserver (https://microreact.org/, last accessed July 2021).

### (f) Analysis of the core genome and tree of *Escherichia coli*

The pangenome, core genome and the tree of *E. coli* were computed with PanACoTA [23]. Briefly, the genomes of *E. coli* in our dataset were retrieved, filtered to remove very closely related genomes (MASH distance less than 0.0001), and then clustered using mmseqs2 with a minimal threshold of 80% identity in protein sequences. Within the pangenome of these 657 genomes there were 2107 families present in more than 90% of the genomes, which make the core genome. The core genome was aligned (for families with not more than a single gene per genome) using mafft-linsi, rendering a multiple alignment with 2 069 583 positions that were used to build a phylogenetic tree by FastTree 2.1 with model JC [24]. The phylogroup of each *E. coli* genome was determined with an approach similar to the one of ClermontTyping method [45]. The position of P4-like elements in *E. coli* was computed in relation to the core genome. The genes of the core genome were positioned as in the first sequenced genome of *E. coli* (strain MG1655). For each P4-like element, we identified the immediately upstream gene family of the core genome in the focal genome. If the core gene family has an ordered position in MG1655 of *N*, we noted *N* + 1 for the position of the P4-like element. We then plotted the histogram of the positions.

## Data accessibility

The bacterial and phage genomes, as well as most profiles used to detect the core components of P4, are publicly available. The custom profile corresponding to sid is included as the electronic supplementary material. The code used to identify the P4-like elements in bacterial genomes is available at https://gitlab.pasteur.fr/jsousa/p4finder.

## References

[1] Penadés JR, Christie GE. 2015 The phage-inducible chromosomal islands: a family of highly evolved molecular parasites. Annu. Rev. Virol. **2**, 181-201. (10.1146/annurev-virology-031413-085446) 26958912

[2] Christie GE, Dokland T. 2012 Pirates of the Caudovirales. Virology **434**, 210-221. (10.1016/j.virol.2012.10.028) 23131350 PMC3518693

[3] Lindqvist BH, Dehò G, Calendar R. 1993 Mechanisms of genome propagation and helper exploitation by satellite phage P4. Microbiol. Rev. **57**, 683-702. (10.1128/mr.57.3.683-702.1993) 8246844 PMC372931

[4] Fillol-Salom A, Martínez-Rubio R, Abdulrahman RF, Chen J, Davies R, Penadés JR. 2018 Phage-inducible chromosomal islands are ubiquitous within the bacterial universe. ISME J. **12**, 2114-2128. (10.1038/s41396-018-0156-3) 29875435 PMC6092414

[5] Seed KD, Lazinski DW, Calderwood SB, Camilli A. 2013 A bacteriophage encodes its own CRISPR/Cas adaptive response to evade host innate immunity. Nature **494**, 489-491. (10.1038/nature11927) 23446421 PMC3587790

[6] McKitterick AC, Hays SG, Johura F-T, Alam M, Seed KD. 2019 Viral satellites exploit phage proteins to escape degradation of the bacterial host chromosome. Cell Host Microbe **26**, 504-514. (10.1016/j.chom.2019.09.006) 31600502 PMC6910227

[7] Moura de Sousa JA, Pfeifer E, Touchon M, Rocha EPC. 2021 Causes and consequences of bacteriophage diversification via genetic exchanges across lifestyles and bacterial taxa. Mol. Biol. Evol. **38**, 2497-2512. (10.1093/molbev/msab044) 33570565 PMC8136500

[8] Novick RP, Ram G. 2017 Staphylococcal pathogenicity islands: movers and shakers in the genomic firmament. Curr. Opin. Microbiol. **38**, 197-204. (10.1016/j.mib.2017.08.001) 29100762 PMC5884141

[9] Rousset F, Dowding J, Bernheim A, Rocha EPC, Bikard D. 2021 Prophage-encoded hotspots of bacterial immune systems. *bioRxiv* 2021.01.21.427644. (10.1101/2021.01.21.427644)

[10] O'Hara BJ, Barth ZK, McKitterick AC, Seed KD. 2017 A highly specific phage defense system is a conserved feature of the *Vibrio cholerae* mobilome. PLoS Genet. **13**, e1006838. (10.1371/journal.pgen.1006838) 28594826 PMC5481146

[11] Briani F, Dehò G, Forti F, Ghisotti D. 2001 The plasmid status of satellite bacteriophage P4. Plasmid **45**, 1-17. (10.1006/plas.2000.1497) 11319927

[12] Dehò G, Ghisotti D, Aland P, Zangrossi S, Borrello MG, Sironi G. 1984 Plasmid mode of propagation of the genetic element P4. J. Mol. Biol. **178**, 191-207. (10.1016/0022-2836(84)90139-6) 6492154

[13] Liu T, Renberg SK, Haggård-Ljungquist E. 1997 Derepression of prophage P2 by satellite phage P4: cloning of the P4 epsilon gene and identification of its product. J. Virol. **71**, 4502-4508. (10.1128/JVI.71.6.4502-4508.1997) 9151842 PMC191670

[14] Halling C, Calendar R. 1990 Bacteriophage P2 ogr and P4 delta genes act independently and are essential for P4 multiplication. J. Bacteriol. **172**, 3549-3558. (10.1128/JB.172.7.3549-3558.1990) 2193911 PMC213327

[15] Ziegelin G, Scherzinger E, Lurz R, Lanka E. 1993 Phage P4 alpha protein is multifunctional with origin recognition, helicase and primase activities. EMBO J. **12**, 3703-3708. (10.1002/j.1460-2075.1993.tb06045.x) 8253092 PMC413647

[16] Shore D, Deho G, Tsipis J, Goldstein R. 1978 Determination of capsid size by satellite bacteriophage P4. Proc. Natl Acad. Sci. USA **75**, 400-404. (10.1073/pnas.75.1.400) 272656 PMC411256

[17] Isaksen ML, Rishovd ST, Calendar R, Lindqvist BH. 1992 The polarity suppression factor of bacteriophage P4 is also a decoration protein of the P4 capsid. Virology **188**, 831-839. (10.1016/0042-6822(92)90538-Z) 1585650

[18] Kizziah JL, Rodenburg CM, Dokland T. 2020 Structure of the capsid size-determining scaffold of ‘satellite’ bacteriophage P4. Viruses **12**, 953. (10.3390/v12090953) PMC7552001 32867300

[19] Dokland T. 2019 Molecular piracy: redirection of bacteriophage capsid assembly by mobile genetic elements. Viruses **11**, 1003. (10.3390/v11111003) PMC6893505 31683607

[20] Kirby JE, Trempy JE, Gottesman S. 1994 Excision of a P4-like cryptic prophage leads to Alp protease expression in *Escherichia coli*. J. Bacteriol. **176**, 2068-2081. (10.1128/JB.176.7.2068-2081.1994) 7511583 PMC205313

[21] Inouye S, Sunshine M, Six E, Inouye M. 1991 Retronphage phi R73: an *E. coli* phage that contains a retroelement and integrates into a tRNA gene. Science **252**, 969-971. (10.1126/science.1709758) 1709758

[22] Bobay L-M, Touchon M, Rocha EPC. 2014 Pervasive domestication of defective prophages by bacteria. Proc. Natl Acad. Sci. USA **111**, 12 127-12 132. (10.1073/pnas.1405336111) PMC4143005 25092302

[23] Perrin A, Rocha EPC. 2021 PanACoTA: a modular tool for massive microbial comparative genomics. NAR Genom. Bioinform. **3**, Iqaa106. (10.1093/nargab/lqaa106) PMC7803007 33575648

[24] Price MN, Dehal PS, Arkin AP. 2010 FastTree 2—approximately maximum-likelihood trees for large alignments. PLoS ONE **5**, e9490. (10.1371/journal.pone.0009490) 20224823 PMC2835736

[25] Goldstein R, Sedivy J, Ljungquist E. 1982 Propagation of satellite phage P4 as a plasmid. Proc. Natl Acad. Sci. USA **79**, 515-519. (10.1073/pnas.79.2.515) 7043461 PMC345774

[26] Konstantinidis KT, Tiedje JM. 2005 Genomic insights that advance the species definition for prokaryotes. Proc. Natl Acad. Sci. USA **102**, 2567-2572. (10.1073/pnas.0409727102) 15701695 PMC549018

[27] Iwabe N, Kuma K, Hasegawa M, Osawa S, Miyata T. 1989 Evolutionary relationship of archaebacteria, eubacteria, and eukaryotes inferred from phylogenetic trees of duplicated genes. Proc. Natl Acad. Sci. USA **86**, 9355-9359. (10.1073/pnas.86.23.9355) 2531898 PMC298494

[28] La Scola B et al*.* 2008 The virophage as a unique parasite of the giant mimivirus. Nature **455**, 100-104. (10.1038/nature07218) 18690211

[29] Matos RC, Lapaque N, Rigottier-Gois L, Debarbieux L, Meylheuc T, Gonzalez-Zorn B, Repoila F, de Lopes M, Serror P. 2013 *Enterococcus faecalis* prophage dynamics and contributions to pathogenic traits. PLoS Genet. **9**, e1003539. (10.1371/journal.pgen.1003539) 23754962 PMC3675006

[30] Asadulghani M, Ogura Y, Ooka T, Itoh T, Sawaguchi A, Iguchi A, Nakayama K, Hayashi T. 2009 The defective prophage pool of *Escherichia coli* O157: prophage–prophage interactions potentiate horizontal transfer of virulence determinants. PLoS Pathog. **5**, e1000408. (10.1371/journal.ppat.1000408) 19412337 PMC2669165

[31] Casjens SR, Grose JH. 2016 Contributions of P2- and P22-like prophages to understanding the enormous diversity and abundance of tailed bacteriophages. Virology **496**, 255-276. (10.1016/j.virol.2016.05.022) 27372181 PMC4969182

[32] Banerjee R, Nath S, Ranjan A, Khamrui S, Pani B, Sen R, Sen U. 2012 The first structure of polarity suppression protein, Psu from Enterobacteria phage P4, reveals a novel fold and a knotted dimer. J. Biol. Chem. **287**, 44 667-44 675. (10.1074/jbc.M112.423202) PMC3531781 23150672

[33] Roux S, Enault F, Hurwitz BL, Sullivan MB. 2015 VirSorter: mining viral signal from microbial genomic data. PeerJ **3**, e985. (10.7717/peerj.985) 26038737 PMC4451026

[34] Eddy SR. 2011 Accelerated profile HMM searches. PLoS Comput. Biol. **7**, e1002195. (10.1371/journal.pcbi.1002195) 22039361 PMC3197634

[35] Oliveira PH, Touchon M, Cury J, Rocha EPC. 2017 The chromosomal organization of horizontal gene transfer in bacteria. Nat. Commun. **8**, 841. (10.1038/s41467-017-00808-w) 29018197 PMC5635113

[36] Steinegger M, Söding J. 2017 MMseqs2 enables sensitive protein sequence searching for the analysis of massive data sets. Nat. Biotechnol. **35**, 1026-1028. (10.1038/nbt.3988) 29035372

[37] Huerta-Cepas J et al*.* 2019 eggNOG 5.0: a hierarchical, functionally and phylogenetically annotated orthology resource based on 5090 organisms and 2502 viruses. Nucleic Acids Res. **47**, D309-D314. (10.1093/nar/gky1085) 30418610 PMC6324079

[38] Grazziotin AL, Koonin EV, Kristensen DM. 2017 Prokaryotic virus orthologous groups (pVOGs): a resource for comparative genomics and protein family annotation. Nucleic Acids Res. **45**, D491-D498. (10.1093/nar/gkw975) 27789703 PMC5210652

[39] Pfeifer E, Moura de Sousa JA, Touchon M, Rocha EPC. 2021 Bacteria have numerous distinctive groups of phage–plasmids with conserved phage and variable plasmid gene repertoires. Nucleic Acids Res. **49**, 2655-2673. (10.1093/nar/gkab064) 33590101 PMC7969092

[40] Katoh K. 2002 MAFFT: a novel method for rapid multiple sequence alignment based on fast Fourier transform. Nucleic Acids Res. **30**, 3059-3066. (10.1093/nar/gkf436) 12136088 PMC135756

[41] Dress AW, Flamm C, Fritzsch G, Grünewald S, Kruspe M, Prohaska SJ, Stadler PF. 2008 Noisy: identification of problematic columns in multiple sequence alignments. Algorithms Mol. Biol. **3**, 7. (10.1186/1748-7188-3-7) 18577231 PMC2464588

[42] Nguyen L-T, Schmidt HA, von Haeseler A, Minh BQ. 2015 IQ-TREE: a fast and effective stochastic algorithm for estimating maximum-likelihood phylogenies. Mol. Biol. Evol. **32**, 268-274. (10.1093/molbev/msu300) 25371430 PMC4271533

[43] Dereeper A et al*.* 2008 Phylogeny.fr: robust phylogenetic analysis for the non-specialist. Nucleic Acids Res. **36**, W465-W469. (10.1093/nar/gkn180) 18424797 PMC2447785

[44] Argimón S et al*.* 2016 Microreact: visualizing and sharing data for genomic epidemiology and phylogeography. Microb. Genom. **2**, e000093. (10.1099/mgen.0.000093) 28348833 PMC5320705

[45] Clermont O, Christenson JK, Denamur E, Gordon DM. 2013 The clermont *Escherichia coli* phylo-typing method revisited: improvement of specificity and detection of new phylo-groups: a new *E. coli* phylo-typing method. Environ. Microbiol. Rep. **5**, 58-65. (10.1111/1758-2229.12019) 23757131

