# Correction: ‘To catch a hijacker: abundance, evolution and genetic diversity of P4-like bacteriophage satellites’ (2022), by Moura de Sousa *et al*.

**DOI:** 10.1098/rstb.2025.0254

**Published:** 2025-08-21

**Authors:** Jorge Moura de Sousa

**Affiliations:** ^1^ Institut Pasteur, Université de Paris, CNRS, UMR3525, Microbial Evolutionary Genomics, Paris 75015, France

*Phil. Trans. R. Soc. B* **377**: 20200475 (published online 17 January 2022) (http://doi.org/10.1098/rstb.2020.0475)

The authors wish to correct an error found in one of the published figure panels (figure 1D). This refers to the position of integrated P4-like elements relative to persistent genes. There was an error in manipulating the data on the order of bacterial core genes, which led the authors to erroneously infer that P4-like satellites are integrated at many different locations of the bacterial genomes, when they are actually present at six specific genomic regions (see corrected figure 1D below).

Although this amendment does not change the results and main conclusions of the article, the authors wish to provide the corrected information.

Changes:

1. Figure 1D should appear as below, with an updated caption.

**Figure 1D.**
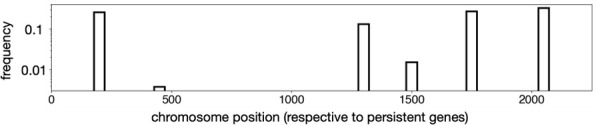
Position of the P4-like element along the chromosome of *E. coli*. The core genes of the species were ordered and numbered in function of the position of the gene in the chromosome of strain MG1655, from 1 to 2099. Positions between core genes define genome intervals. The position indicates the interval where the P4-like element was identified. Hence, an element integrating the same region across genomes would have the same relative position.

2. Some text in §2 is updated to reflect the above.

*Original text* (§2a*)*: To confirm that their prevalence is not the result of a single ancestral infection, we analysed the positions of P4-like elements in the chromosomes of the species (see Methods). We located the elements in relation to the positions of the neighbouring core genes and found that P4-like elements are scattered across the *Escherichia coli* chromosome (figure 1D). The dispersion of the elements across the phylogenetic tree and across the chromosome shows that these elements have proliferated by horizontal transfer across the species.

*Modified text* (§2a*)*: To confirm that their prevalence is not the result of a single ancestral infection, we analysed the positions of P4-like elements in the chromosomes of the species (see §4). We located the elements in relation to the positions of the neighbouring core genes and found that P4-like elements are integrated at six different regions in the *E. coli* chromosome (figure 1D). The dispersion of the elements across the phylogenetic tree and across several integration sites in the chromosome shows that these elements have proliferated by horizontal transfer across the species.

